# Lysophosphatidic Acid Mediates Myeloid Differentiation within the Human Bone Marrow Microenvironment

**DOI:** 10.1371/journal.pone.0063718

**Published:** 2013-05-16

**Authors:** Denis Evseenko, Brooke Latour, Wade Richardson, Mirko Corselli, Arineh Sahaghian, Sofie Cardinal, Yuhua Zhu, Rebecca Chan, Bruce Dunn, Gay M. Crooks

**Affiliations:** 1 University of California Los Angeles (UCLA), Department of Orthopaedic Surgery, Los Angeles, California, United States of America; 2 Eli and Edythe Broad Institute for Regenerative Medicine and Stem Cell Research, Los Angeles, California, United States of America; 3 University of California Los Angeles (UCLA), David Geffen School of Medicine, Department of Pathology and Laboratory Medicine, Los Angeles, California, United States of America; 4 University of California Los Angeles (UCLA), Department of Engineering, Los Angeles, California, United States of America; 5 University of California Los Angeles (UCLA), David Geffen School of Medicine, Department of Pathology and Laboratory Medicine, Los Angeles, California, United States of America; University of Manitoba, Canada

## Abstract

Lysophosphatidic acid (LPA) is a pleiotropic phospholipid present in the blood and certain tissues at high concentrations; its diverse effects are mediated through differential, tissue specific expression of LPA receptors. Our goal was to determine if LPA exerts lineage-specific effects during normal human hematopoiesis. In vitro stimulation of CD34+ human hematopoietic progenitors by LPA induced myeloid differentiation but had no effect on lymphoid differentiation. LPA receptors were expressed at significantly higher levels on Common Myeloid Progenitors (CMP) than either multipotent Hematopoietic Stem/Progenitor Cells (HSPC) or Common Lymphoid Progenitors (CLP) suggesting that LPA acts on committed myeloid progenitors. Functional studies demonstrated that LPA enhanced migration, induced cell proliferation and reduced apoptosis of isolated CMP, but had no effect on either HSPC or CLP. Analysis of adult and fetal human bone marrow sections showed that PPAP2A, (the enzyme which degrades LPA) was highly expressed in the osteoblastic niche but not in the perivascular regions, whereas Autotaxin (the enzyme that synthesizes LPA) was expressed in perivascular regions of the marrow. We propose that a gradient of LPA with the highest levels in peri-sinusoidal regions and lowest near the endosteal zone, regulates the localization, proliferation and differentiation of myeloid progenitors within the bone marrow marrow.

## Introduction

Lysophosphatidic acid (LPA) is a phospholipid that mediates a myriad of biological actions, including cell proliferation, migration, and survival. LPA species are detectable in biological samples such as plasma and saliva and are secreted by activated platelets as a major growth factor in serum [1]. Albumin binds LPA and protects it from degradation [2]; thus high levels of LPA in serum create a challenge when testing the effect of LPA on hematopoiesis using either in vitro or in vivo assays. Autotaxin (ATX) is the key LPA producing enzyme in plasma and eukaryotic tissues, mediating removal of choline from lysophosphatidylcholine [3]. Cell membrane lipid phosphate phosphatases (PPAP), most importantly PPAP2A, attenuate the activity of LPA by dephosphorylation [4]. The pleiotropic effects described for LPA are in part due to differential expression patterns of LPA receptors (LPAR_1_-LPAR_6_) within different tissues [5].

Several studies have demonstrated a role for sphingosine-1 phosphate (S1P), a lipid structurally related to LPA, in increasing engraftment by augmenting signaling through CXCR4 in response to stromal derived growth factor-1 (SDF-1) [6]. However, little is known about the role of LPA signaling during hematopoietic differentiation. A recent study demonstrated LPAR3 is essential for the induction of erythropoiesis [7], and another showed that LPA enhances migration of murine lin-sca-1+ckit+ cells, a population that includes hematopoietic stem cells and early progenitors [8]. Our goal was to investigate the role of LPA during lineage commitment of human hematopoietic progenitors.

## Materials and Methods

### Isolation of Human Progenitor Populations

Umbilical cord blood (CB) was collected from normal deliveries, according to guidelines approved by the University of California Los Angeles Investigational Review Board. Enrichment of CD34+ cells was performed using the magnetic-activated cell sorting system (Miltenyi Biotec, Auburn, CA). For fluorescence-activated cell-sorting (FACS) sorting, CD34+ enriched cells were incubated with the following anti-human–specific monoclonal antibodies: CD34 PerCP-Cy5.5, CD38 PE-Cy7, CD123 (interleukin-3 receptor alpha) PE, CD45RA PE-Cy5, FITC-labeled lineage-specific antibodies: CD2, CD3, CD4, CD8, CD7, CD10, CD11b, CD14, CD19, CD56, and glycophorin A (Gly A); all from Becton Dickinson, San Jose, CA). An unstained (no antibody) control was used to define negative gates. The following, previously published immunophenotypic definitions were used to isolate myeloid progenitors from thawed CB CD34+ enriched cells by FACS: CD34+CD38-lin-CD45RA-CD123lo (CMP) [9], CD34+CD10+lin- CLP [10] and CD34+CD38-lin- hematopoietic stem/progenitor cells (HSPC) [11]. Sorting was performed on a FACSAria (Becton Dickinson) equipped with five lasers (355, 405, 488, 561, and 633 nm). Isolated populations were analyzed by FACS to assess post sort purity. For all FACS sorted populations ∼ 95–99% purity was achieved based on re-analysis.

### Hematopoietic Cultures

Cocultivation on the murine stromal line OP9 [12]was used to test for B lymphoid and myeloid differentiation. Freshly sorted CD34+ cord blood cells (500–1500 cells) were seeded onto established non-irradiated OP9 stromal cells (American Type Culture Collection, Manassas, VA) in 96-well or 48-well flat-bottomed plates. Cells were grown in a modified medium (DMEM/F12, Invitrogen, Carlsbad, CA) supplemented with 5% fetal bovine serum (Invitrogen, Carlsbad, CA) treated with charcoal to remove LPA, 50 µM 2-mercaptoethanol (Sigma-Aldrich), penicillin/streptomycin (Gemini Bio Products, Calabasas, CA), IL-7 (5 ng/mL, R&D Systems, Minneapolis, MN), Flt3 ligand (FL, 5 ng/mL, R&D), and thrombopoietin (TPO, 5 ng/mL, R&D). This cytokine combination is permissive for both lymphoid (B-cells) and myeloid (monocytic, granulocytic and megakaryocytic) lineages. Every 3 days thereafter, half the medium was replaced with fresh medium. Lysophosphatidic acid 18∶1 Oleoyl-LPA (Tocris Bioscience, MA) was reconstituted in 70% ethanol and added to the fresh culture medium at final concentrations 0.1, 1 or 10 uM initially to determine optimal dose response. All subsequent experiments used a concentration of 1 µM LPA. Cells were cultured for 4 weeks followed by harvesting, immunostaning with fluorochrome labeled antibodies and immunophenotypic analysis of cultured cells. Sphingosine-1-Phosphate and Ki-16425 were purchased from Tocris Bioscience and reconstituted in 4% fatty acid free albumin (Sigma Aldrich, St Louis, MO) solution in phosphate buffered saline or 70% ethanol respectively following the manufacturer’s instructions.

### Immunophenotypic Analysis of Cultured Cells

**Figure 1:**
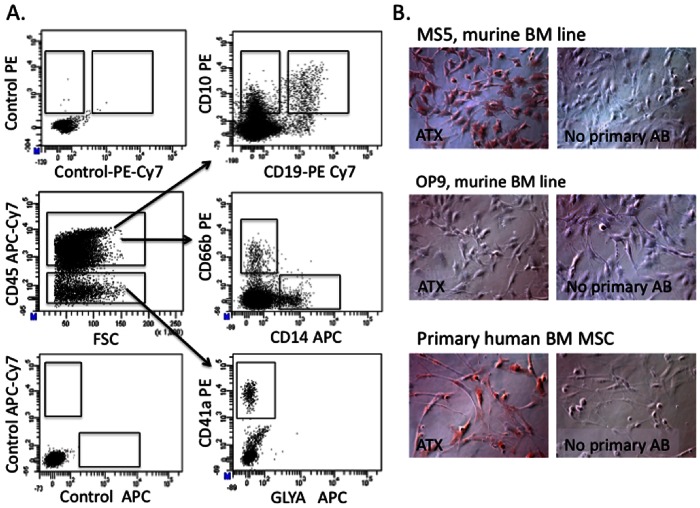
In vitro system for differentiation of hematopoietic cells. **A.** In vitro differentiation of un-fractionated CD34+ progenitors co-cultured on a stromal monolayer in medium containing 5% LPA-depleted serum in the presence of thrombopoietin, Flt3 Ligand and IL-7, cytokines that allow generation of both myeloid (CD45+CD14+ monocytes, CD45+CD66b granulocytes and CD45^neg^CD41a+ megakaryocytes) and B lymphoid cells: CD45+CD10+ CD19neg or CD45+CD10+CD19+. Control panel represents unstained cells. **B.** Autotaxin protein expression in commonly used stromal lines: MS5, OP9 and human bone marrow-derived mesenchymal stromal cells. Positive signal is shown in brown color (DAB). Magnification 20X. Images were acquired using the Zeiss Axiovision software version 4.8 Carl Zeiss Microscope (Carl Zeiss, Germany).

FACS analysis of cultured cells was performed on an LSR II instrument (Becton Dickinson) by direct immunofluorescence staining with human specific monoclonal antibodies after incubation in 1.2% human intravenous immunoglobulin (IVIG; Cutter, Berkley, CA). Lineage-specific differentiation was determined using the following antibodies: CD45-APC Cy7, CD34-PE Cy7, CD41a-PE Cy5, CD66b-PE, GlyA-APC or -PE, CD19-PE, APC or Percp-Cy5.5, and CD14 PE or FITC (all from Becton Dickinson). The following immunophenotypes were used to identify terminally differentiated lymphoid and myeloid cells from culture: monocytes (CD14+CD45+), granulocytes (CD66b+CD45+), megakaryocytes (CD41a+CD45-GLYA-) and B-cells (CD19+CD45+) (Fig. 1A). For long term (4 week) culture experiments, the total number of cells per well in each condition was determined by trypan blue microscopy, and the number of differentiated cells was calculated based on % of each lineage phenotype by FACS multiplied to a total cell number in each well.

### Cell Migration Experiments

Migration assays were carried out in 24 well Transwell plates from Costar with 6.5 mm diameter and 8.0 µm pore size. Freshly sorted CMP, CLP or HSPC (1,000 cells each) were seeded into the upper chamber in DMEM/F12 supplemented with 5% charcoal treated serum with no growth factors in the presence or absence of LPA (1 uM) in the lower chamber. Migration was assessed based on the number of total cells on the bottom of the lower chamber after 12 hours, determined separately for each cell type using bright field microscopy. Independent experiments were carried out using progenitor populations isolated from 3 different donors.

### Cell Proliferation and Apoptosis Analyses

Freshly sorted CMP, CMP or HSPC were seeded into 48 well plates (5,000 cells per well) on OP9 cells, with DMEM/supplemented with 5% charcoal treated serum, with no growth factors in the presence or absence of LPA (1 uM) and cultured for 48 hours without medium change to measure the effect of LPA on proliferation and apoptosis. Prior to harvesting, cells were incubated with bromodeoxyuridine (BrdU) (10 uM) for 30 minutes. Harvested cells were fixed, permeabilized, and stained with FITC or APC conjugated antibody against BrdU. Unstained cells were used to set negative gates. Apoptosis rates in progenitor populations wasere assessed using FACS-based Annexin V assay (BD Bioscience). Equal numbers (3000 of CD45 gated hematopoietic cells recovered from culture) of each population was analyzed for BrdU incorporation or Annexin V binding using FACS analysis.

### Immunocytochemistry

Specimens of adult sponge bone (3 individual specimens) were provided by the UCLA Translation Pathology Core Laboratory. All specimens were from patients with no hematopoietic disorders. Fetal bones (16–18 weeks of pregnancy) were obtained from Novogenix (Los Angeles, CA) fixed in 4% paraformaldehyde (Sigma-Aldrich, St. Lois, MO, in PBS). Fixed tissues were embedded in paraffin, sectioned and subjected to histological immunohistochemical analyses. The murine stromal cell lines MS5 and OP9 (American Type Culture Collection (ATCC, Rockville, MD, USA) or primary human bone marrow derived mesenchymal stromal cells (passage 2–3) (AllCells Inc, Emeryville CA) were seeded into chamber slides (BD Bioscience) in DMEM/F12 (Invitrogen) medium supplemented with 20% fetal bovine serum. The next day, cells were fixed with 4% paraformaldehyde and subjected immunohistochemical analysis. Polyclonal antibodies against autotaxin, PPAP2a and CD146 were purchased from Abcam Inc. (Cambridge, MA) Secondary horse radish peroxidase (HRP) conjugated IMPRESS anti-rabbit and anti-mouse antibodies and 3, 3′-diaminobenzidine (DAB) substrate (Vector Labs) were used for the visualization of positively labeled regions. Images were acquired using the Zeiss Axiovision software version 4.8 Carl Zeiss Microscope (Carl Zeiss, Germany) equipped with ApoTome.2: Modules for Axio Imager.2 and Axio Observer with 40x (1.3 numerical aperture (NA)) and 63x (1.4 NA) oil-immersion objectives.

### Quantitative Real-time PCR

Total RNA was extracted from cells (∼5,000 sorted cells were used for each population) using the RNeasy Micro Kit, and converted to cDNA using the Omniscript RT Kit (kits were from Qiagen Sciences, Maryland, USA). Total RNA concentration for all samples was within 5–20 ng/ml range as determined by Nanodrop analysis. No template amplification was carried out prior to cDNA synthesis.

Next, SYBR Green RT-PCR amplification and detection was performed using an ABI Prism 7900 HT (Applied Biosystems) as previously described. The comparative C_t_ method for relative quantification (2^−ΔΔCt^) was used to quantitate gene expression according to Applied Biosystems’ recommendations [*7900 HT Real-Time fast and SDS enterprise and database user guide*]. Expression of target genes was normalized to the level of the house-keeping gene RPL-7 and expressed relative to a calibrator (sample in each set with lowest expression). All primer sequences were obtained from Harvard University Primer Bank (http://pga.mgh.harvard.edu/primerbank) Primer sequences used for QPCR are available on request.

## Results

### LPA Stimulates Myeloid Differentiation

**Figure 2:**
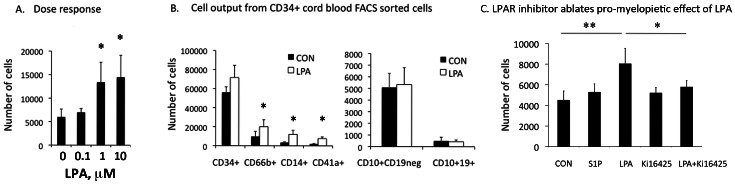
LPA stimulates generation of myelopoietic lineages from cord blood CD34+ progenitor cells. **A.** Dose response of CD34+ cord blood FACS sorted cells to increasing concentration (0.1, 1, 10 uM of LPA). Detection of CD14+ monocytes was used as readout of activity. Mean ± standard deviation (SD), Mean 3 *p<0.05 compared to control cells. **B.** LPA stimulated generation of myeloid (monocytes, granulocytes and megakaryocytes), but not lymphoid (B-cell) differentiation from CD34+ cells. Freshly sorted CB CD34+ cells were cultured on OP9 stroma for 4 weeks in medium supplemented with 5% LPA-depleted (charcoal treated) serum and growth factor combinations permissive for both myeloid and lymphoid differentiation in the absence (CON = control) or presence of LPA (1 uM). The total number of cells per well in each condition was determined by counting in hematocytometer, and the number of cells of each immunophenotype (shown on the y-axis) was calculated based on % of each lineage phenotype by FACS multiplied to total cell number in each well. Shown is Mean ± standard deviation (SD), N = 4 independent experiments, *p<0.05. **C.** Stimulatory effects of LPA on myelopoiesis can be ablated using LPA receptor antagonist Ki16425. Myelopoietic differentiation of CD34+ cord blood cells was assessed by the generation of CD14+CD45+ monocytes at 7 days of culture. Concentration of tested compounds: LPA and S1P –1 uM, Ki16425–5 uM. Cord blood samples from 4 donors were analyzed independently and results shown as Mean ± SD. ** P<0.01, * P<0.05. CON = Control.

To dissect the role of LPA on human hematopoiesis we began by analyzing how LPA affects differentiation of un-fractionated CD34+ progenitors, a heterogeneous population that includes hematopoietic stem cells and multi-lineage progenitors (HSPC), as well as myeloid and lymphoid-committed progenitors. CD34+ cells were co-cultured on a stromal monolayer in medium containing 5% LPA-depleted serum [13], with or without addition of exogenous LPA and in the presence of thrombopoietin, Flt3 Ligand and IL-7, cytokines that allow generation of both myeloid and B lymphoid cells (Fig. 1A, 2A). To determine the optimal stromal layer for these experiments, we analysed expression of ATX, the enzyme which mediates synthesis of LPA, in 3 stromal cell types commonly used for hematopoietic cell support in vitro: OP9 [12], MS5 stroma [14] and primary bone marrow derived mesenchymal stromal cells (BM MSC). OP9 demonstrated very low levels of ATX suggesting that this line has minimal production of endogenous LPA in culture (Fig. 1B). In contrast to OP9, both MS5 and primary BM MSC showed readily detectable levels of ATX protein expression (Fig. 1B). Based on the pattern of ATX production in the tested stromal lines we used OP9 cells for all our co-culture experiments. We next tested the effect of different LPA concentrations (0.1, 1 and 10 uM) on lineage differentiation from CD34+ cord blood cells. Generation of CD14+ monocytes was significantly enhanced in the presence of 1 and 10 mM of LPA while 0.1 uM of LPA had little or no effect (Fig. 2A). LPA was thus used at a concentration of 1 uM for further detailed experimentation.

The addition of LPA in culture generated significantly more myeloid cells (CD66b+ granulocytes and CD14+ monocytes) and CD41a+ megakaryocytes from CD34+ cells compared to controls (otherwise identical culture conditions without LPA added). In contrast B lymphocyte output was not altered by the presence of LPA, and the number of CD34+ progenitors persisting after 4 weeks was not significantly changed (Fig. 2B). LPA induced similar effects on CD34+ cells isolated from human bone marrow to those from cord blood (not shown). These data suggest that LPA has a selective and significant effect on myeloid differentiation.

We next explored the specificity of observed stimulatory effects of LPA on differentiation of CD34+ hematopoietic progenitors. Addition of the sphingosine-1-phosphate, a lipid molecule structurally similar to LPA, did not show any significant pro-myelopoietic effects (Fig. 2C). LPA receptor antagonist Ki-16425 almost completely abolished stimulatory effects of LPA on CD34+ cell differentiation further confirming specificity of the LPA mediated stimulation of myelopoiesis (Fig. 2C).

**Figure 3:**
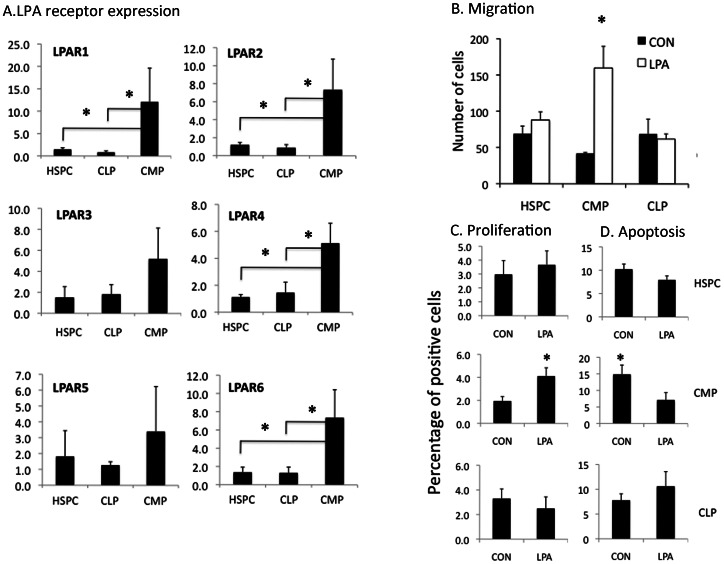
Myeloid progenitors are functional targets of LPA. **A.** LPA receptor mRNA expression in hematopoietic stem-progenitor cells (HSPC), common lymphoid (CLP) and myeloid progenitors (CMP) by qPCR. N = 4 independent experiments; *p<0.05. **B.** Migration in Transwell® experiments in the presence or absence of 18∶1 Oleoyl-LPA, 12 hours after seeding of HSPC, CMP or CLP. **C.** Proliferation of each cell type shown measured by 48 hour BrdU uptake. Y axis shows % of BrdU positive cells) **D.** Apoptosis of each cell type measured by the Annexin V assay (Y axis-shows the % of Annexin V positive cells). Mean ± SD; N = 3 independent experiments; *p<0.05.

To further understand the mechanisms by which LPA induced myeloid differentiation, we explored LPA receptor expression on specific hematopoietic stem and progenitor populations. Real time RT-PCR was performed on freshly isolated CD34+CD38-lin- cells (enriched for HSPC), CD34+lin-CD45RA-IL3Ra^lo^ common myeloid progenitors (CMP), and CD34+lin-CD10+ common lymphoid progenitors (CLP) (a population with predominantly B cell potential [15] (Fig. 3A). Expression of all six LPA receptors was higher in CMP than either HSPC or CLP (p<0.05 for LPAR 1,2,4 and 6).

### LPA Stimulates the Migration, Proliferation and Survival of Myeloid but not Lymphoid Progenitors

Functional studies were next performed to define the effects of LPA stimulation on lineage-specific progenitors. In a transwell migration assay, LPA selectively stimulated the migratory potential of CMP but had no detectable effect on either HSPC or lymphoid progenitors (Fig. 3B). Cell proliferation after 48 hours of LPA stimulation (measured by BrdU uptake) was significantly increased in CMP but not HSPC or CLP (Fig. 3C). In addition, LPA reduced apoptosis of CMP but had no effect on either HSPC or CLP (Fig. 3D). Of note, all migration assays were performed in stroma-free conditions and therefore represented direct effects of LPA on hematopoietic progenitors.

### ATX and PPAP2A are Differentially Expressed in Human Bone Marrow

Published data suggest that the hematopoietic niche is spatially organized between the endosteal region and the sinusoidal perivascular zones [16], [17], [18]. As myeloid and lymphoid commitment from HSPC is regulated in large part by differential signals emanating from the microenvironment, it is plausible that these lineages develop in spatially distinct compartments within the bone marrow. Indeed, some studies have indicated that myeloid cells accumulate in perisinusoidal regions before they enter systemic circulation [16], [18], and others that pre-B cells migrate and accumulate in close proximity to osteoblasts [17], The mechanisms controlling this separation of lymphoid and myeloid cells in the bone marrow niche are not known.

In view of our findings that LPA induces migration and growth specifically of myeloid progenitors, we hypothesized that LPA might play a role in the compartmentalization of the bone marrow niche. As LPA levels cannot be assayed in situ directly, we studied the expression and the spatial localization within adult bone marrow of ATX and PPAP2A, the enzymes responsible for synthesis and degradation of LPA respectively.

**Figure 4:**
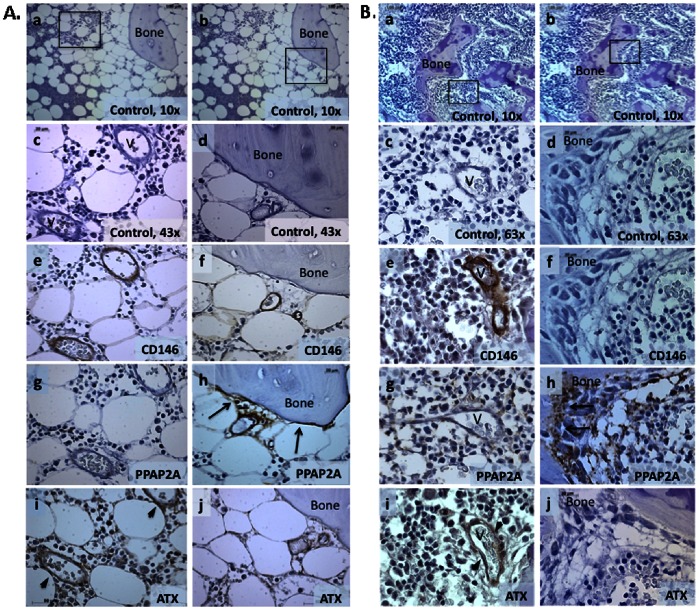
Spatial distribution of autotaxin and PPAP2A in adult bone marrow. **A.** Adult Bone marrow. Low power (10x) unstained sections showing two different focal plans (a, b). (A–c,e,g,i) shows high power views (43x) of boxed area in (a). (A–d,f,h,j) show high power views of boxed area in (b). (Aa–d) controls stained with secondary antibody only, (A–e, f) CD146 detection of perivascular cells, (A–g, h) PPAP2A expression in endosteal region (arrows), but not perivascular cells, (A–i, j) ATX expression limited to perivascular cells (arrow heads). V = vascular space. **B.** Fetal Bone marrow. Consistent with the expression pattern of PPAP2A in adult BM, PPAP2A immunoreactivity in fetal BM was predominantly located in bone surfaces lining osteoblasts (arrows), ATX expression limited to perivascular cells (arrow heads) (Fig. 2B–g, h). Low power views (10x) are shown in a, and b. High power views (63x) of areas boxed in panels at left are shown in Bc, e, g, i. High power views (63x) of areas boxed in panels at right are shown in Bd, f, h and j. Positive signals in A and B are shown in brown color (DAB). Images were acquired using the Zeiss Axiovision software version 4.8 Carl Zeiss Microscope (Carl Zeiss, Germany) equipped with ApoTome.2: Modules for Axio Imager.2 and Axio Observer with 40x (1.3 numerical aperture (NA)) and 63x (1.4 NA) oil-immersion objectives.

In adult bone marrow, PPAP2A was highly expressed by osteoblasts throughout the endosteal zone (Fig. 4A–h). PPAP2A was largely absent in the perivascular region (Fig. 4A–g) with the exception of vessels near the endosteal zone where perivascular cells form “stromal bridges” with osteoblasts (Fig. 4A–h). Little or no ATX expression was found in the osteoblastic region of adult human bone marrow (Fig. 4A–j). However, ATX was highly expressed by CD146+ perivascular stromal cells of blood vessels (Fig. 4A–i).

To investigate whether the spatial distribution of LPA-generating and -metabolising enzymes was age specific and/or related to bone marrow involution and fat deposition associated with aging, ATX and PPAP2A expression was analysed in human fetal (16–18 week old) bone marrow (Fig. 4B). Consistent with the pattern observed in adult bone marrow, high levels of PPAP2A and no/little ATX expression were seen in the endosteal region (Fig. 4B–h,j), whereas ATX expression was clearly present in perivascular regions of blood vessels (Fig. 4B–i). Thus spatial distribution of ATX and PPAP2A during fetal hematopoiesis is similar to that seen in adult.

## Discussion

Although numerous studies have investigated the hematopoietic stem cell niche, relatively few have been specifically focused on the localisation of more mature hematopoietic cells within the bone marrow. Injection of radiolabelled pre-B cells to mice with severe combined immunodeficiency lacking lymphoid cells demonstrated that most of the injected cells migrated into the bone marrow and homed near osteoblasts [17] selectively occupying microenvironments near the surrounding bone. In contrast, myeloid cells have been described as concentrated in close proximity to sinusoids [16]. Moreover megakaryocytes can invaginate into the luminal space of sinusoids where they give rise to terminally differentiated platelets migrating directly into the circulating blood [18]. The exact mechanisms regulating hematopoietic cell compartmentalisation in the bone marrow niche are not clear. It is plausible to predict that regulatory gradients are needed to separate different hematopoietic lineages in the bone marrow and also provide migration signals for differentiated blood cells prepared to enter systemic blood circulation. The spatial expression of ATX and PPAP2A suggests that the highest levels of LPA in this system will appear in the close proximity to the small blood vessels where LPA molecules diffuse directly from blood plasma. High levels of LPA would also be predicted in perivascular regions near larger microvessels (20–100 mkm in diameter) where ATX is expressed. In close proximity to osteoblasts, especially those located remotely from microvessels, levels of LPA would be expected to be minimal due to the high PPAP2A activity and minimal expression of ATX. S1P, another member of the lysophospholipid family, has previously been demonstrated to play a role in both HSPC and lymphoid compartmentalization [8]; high levels of S1P in peripheral blood compared with the bone marrow tissue create a gradient that promotes migration of B-cell progenitors from the bone marrow to secondary lymphoid organs [19]. Disruption of this gradient abrogates HSPC mobilization following AMD3100 treatment thus proposing a critical role for bioactive lipid gradients in hematopoietic cell migration [20]. The data presented here, identify LPA as a regulator of migration, growth and survival of myeloid progenitors. We propose that LPA provides a novel mechanism through which anatomical partitioning of the bone marrow microenvironment creates spatial regulation of myeloid differentiation during hematopoiesis.
